# Role of B-Cell Activating Factor (BAFF) in Inflammatory Bowel Disease

**DOI:** 10.3390/diagnostics12010045

**Published:** 2021-12-27

**Authors:** Marko Kumric, Piero Marin Zivkovic, Tina Ticinovic Kurir, Josip Vrdoljak, Marino Vilovic, Dinko Martinovic, Andre Bratanic, Ivan Kresimir Lizatovic, Josko Bozic

**Affiliations:** 1 Department of Pathophysiology, University of Split School of Medicine, 21000 Split, Croatia; marko.kumric@mefst.hr (M.K.); piero.zivkovic@gmail.com (P.M.Z.); tticinov@mefst.hr (T.T.K.); josip.vrdoljak@mefst.hr (J.V.); marino.vilovic@mefst.hr (M.V.); dinko.martinovic@mefst.hr (D.M.); 2 Department of Gastroenterology, University Hospital of Split, 21000 Split, Croatia; andrebratanic@net.hr; 3 Department of Endocrinology, Diabetes and Metabolic Diseases, University Hospital of Split, 21000 Split, Croatia; 4 Department of Acute Respiratory Infections, University Hospital for Infectious Diseases Dr. Fran Mihaljevic, 10000 Zagreb, Croatia; lizatovic.i.k@gmail.com

**Keywords:** B-cell activating factor, inflammatory bowel disease, calprotectin, biomarker, irritable bowel syndrome, diagnosis

## Abstract

As early commencement of inflammatory bowel disease (IBD) treatment has been shown to substantially improve outcomes, it is of utmost importance to make a timely diagnosis of this disease. Despite undisputed sensitivity of fecal calprotectin, the most widely accepted IBD biomarker, in discriminating between irritable bowel syndrome (IBS) and IBD, as well as recognized role in monitoring disease activity and response to therapy, perhaps the biggest setback of calprotectin use in IBD is lack of specificity. Therefore, an additional biomarker in IBD is warranted. B-cell activating factor (BAFF), a member of the tumor necrosis factor (TNF) superfamily, recently emerged as a viable candidate for this role. So far, overproduction of BAFF has been observed in various autoimmune diseases, most notably in systemic lupus erythematosus, where BAFF-inhibitor belimumab was approved for treatment. As BAFF levels were also shown to correlate with indices of IBD, in this review we aimed to summarize the current evidence with respect to the role of BAFF in diagnosis and assessing the activity of IBD, as well as putative therapeutic implications that may arise from exploring of this relation.

## 1. Introduction

Inflammatory bowel disease (IBD) is a chronic immunologically-mediated disease at the intersection of complex interactions between genetics, environment and gut microbiota [1,2,3]. The two main representatives of IBD are ulcerative colitis (UC) and Crohn’s disease (CD), each with a set of overlapping, but also with one of distinct clinical, pathological and immunological features. Notably, in the recent years, global prevalence of IBD has increased, as the number of countries adopting “western lifestyle” is on the rise [4].

As early onset of IBD treatment has been shown to substantially improve outcomes, it is of utmost importance to make a timely diagnosis of this disease [5]. In the contemporary clinical setting, diagnosis of IBD is based on epidemiologic, clinical and genetic parameters, and usually requires invasive, not to mention expensive, testing [6,7]. Bearing in mind the effects of COVID-19 on global IBD management in terms of resources and epidemiological measures, it is now even more important to establish an alternative noninvasive biomarker for IBD patients [8]. The main utility of the putative diagnostic biomarker would be to establish a more meticulous stratification of patients that will be referred to invasive modalities for confirmation of IBD diagnosis. Furthermore, disease activity being an important guide in IBD therapeutic approach, it would be beneficial if biomarker levels correlated with disease activity.

As the main crossroad of IBD development seems to lie in impaired inflammatory response, most of the biomarkers are associated with immunological response [9]. Currently, the most widely accepted IBD biomarker is fecal calprotectin, a calcium and zinc binding protein, representing about 60% of soluble proteins of the cytoplasm of granulocytes [10]. Despite undisputed sensitivity in discriminating between irritable bowel syndrome (IBS) and IBD, as well as recognized role in monitoring disease activity and response to therapy, perhaps the biggest setback of calprotectin use in IBD is lack of specificity [11]. Namely, many factors, such as age, diet, exercise, colonic cleansing, and fecal amount of mucus or blood in stools, may influence fecal calprotectin levels [12,13,14]. Therefore, an additional biomarker in IBD is warranted.

B-cell activating factor (BAFF), a member of the tumor necrosis factor (TNF) superfamily, recently emerged as a viable candidate for this role [15]. BAFF is predominantly produced by myeloid cells (monocytes, macrophages, dendritic cells and neutrophils), and its main role is regulation of mature B cell survival and differentiation into antibody-producing plasma cells [16,17]. Overproduction of BAFF has been observed in various autoimmune diseases, most notably in systemic lupus erythematosus (SLE), where BAFF-inhibitor belimumab was approved for treatment [18]. Accordingly, accumulating data suggests that BAFF serum and fecal levels are elevated in patients with IBD [15]. Moreover, data obtained from colonic biopsies from IBD patients further supports involvement of BAFF in IBD pathogenesis. Hence, we aimed to review the current evidence with respect to the role of BAFF in diagnosis and disease activity of IBD, as well as putative therapeutic implications that may arise from exploring of this relation.

## 2. BAFF Biology and Role in Other Diseases

**Figure 1:**
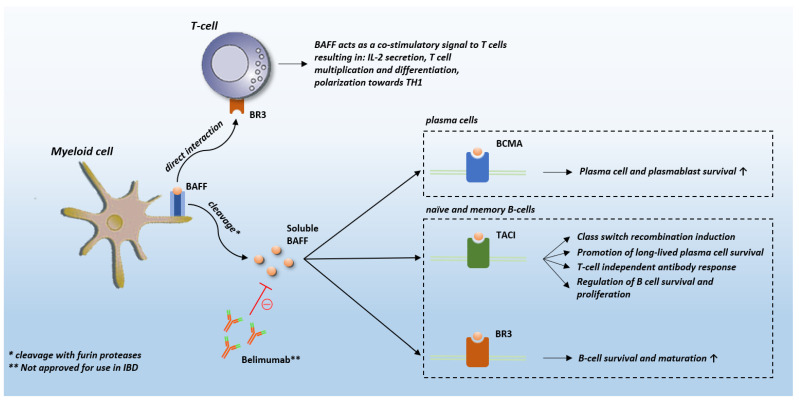
BAFF receptors. Abbreviations: BAFF: B-cell activating factor; BCMA: B cell maturation antigen; TACI: transmembrane activator and calcium-modulating and cyclophilin ligand interactor; BR3: BAFF-receptor; IBD: inflammatory bowel disease; IL-2: interleukin 2.

BAFF is homotrimer transmembrane protein which belongs to the TNF ligand superfamily [19]. Upon cleavage by furin proteases, BAFF is released into circulation as a soluble and biologically active 17-kDa protein [19]. BAFF binds to three distinct receptors, each with distinctive expression patterns based on B cell development stages and related to associated function of each: B cell maturation antigen (BCMA), transmembrane activator and calcium-modulating and cyclophilin ligand interactor (TACI), and BAFF-receptor (BR3) [20,21,22,23]. In the transitional B cell stage BR3 is the predominant receptor on naïve and memory B cells, and to some extent on T-cells, TACI on marginal zone B cells and short-lived plasma cells, whereas BCMA is the principal receptor on long-lived plasma cells (Figure 1) [24]. Under physiological conditions, the main producers of BAFF are myeloid cells (monocytes, macrophages, neutrophils, mast cells, and dendritic cells) [25,26]. BAFF expression can be substantially upregulated upon stimulation with proinflammatory cytokines, bacterial lipopolysaccharide (LPS) and toll-like receptor (TLR) activation [27,28]. Furthermore, non-myeloid cells such as T-cells and B-cells can also produce BAFF [29,30]. Finally, non-hematopoetic cells, such as stromal cells (but many others as well), were also shown to produce BAFF, especially in autoimmune and malignant diseases [31].

It has so far been well-established that BAFF has an important role in the development and function of immune cells. While B-cell function is the primary target of BAFF, other constituents of immune system may be targeted as well. The principal roles of BAFF are promoting of B-cell survival, maturation and regulation of B-cell function. B-cell survival is achieved by decreasing the expression of pro-apoptotic (Bak, Blk, and Bim) molecules, whilst increasing anti-apoptotic (Al, Bcl-2, Bcl-xL, and Mcl-1) molecules [32,33]. Furthermore, BAFF favors the survival of high-affinity B-cell clones and activates class switch recombination of immunoglobulins [34,35]. It has been demonstrated that overexpression of BAFF in mice induces a significant expansion of activated B-cells and marginal zone B-cells, as well as hypergammaglobulinemia and autoantibody production [36,37]. On the other hand, BAFF co-stimulates T-cell activation and proliferation and promotes its differentiation into effector cells [38]. Finally, BAFF promotes activation of both monocytes and dendritic cells [31].

**Figure 2:**
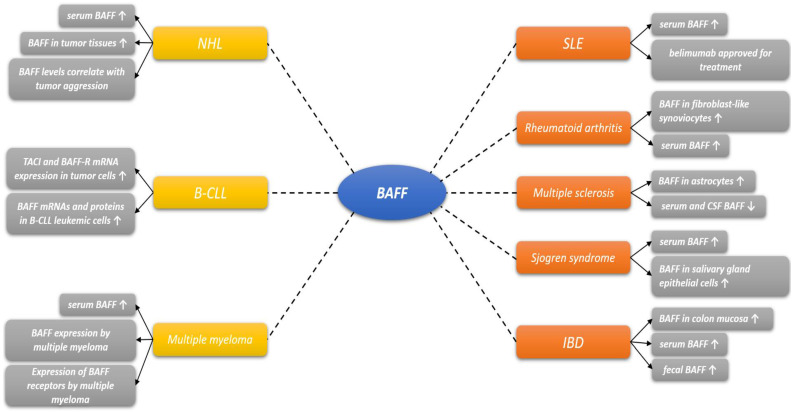
Involvement of BAFF in various diseases. Abbreviations: BAFF-R: B-cell activating factor receptor; BCMA: B cell maturation antigen; TACI: transmembrane activator and calcium-modulating and cyclophilin ligand interactor; CSF: cerebrospinal fluid; NHL: non-Hodgkin lymphoma; B-CLL: B-Cell chronic lymphocytic leukemia; IBD: inflammatory bowel disease.

The first line of evidence to imply involvement of BAFF in autoimmune pathogenesis is the observation that BAFF transgenic mice develop features of autoimmune diseases, most likely owing to increased survival signals to autoreactive B-cells [39]. Apart from substantial amount of data obtained from preclinical experiments, multiple clinical studies observed elevated levels of BAFF in specific tissues associated with the pathogenesis of autoimmune diseases and malignancies (Figure 2). Specifically, BAFF expression has been observed in salivary gland epithelial cells in Sjogren’s syndrome, fibroblast-like synoviocytes in the synovium of patients with rheumatoid arthritis, osteoclasts in patients with multiple myeloma, astrocytes in patients with multiple sclerosis and in gut lavage fluid of patients with food hypersensitivity [40,41,42,43,44]. Hence, it seems that under inflammatory conditions cytokines induce the expression of BAFF by non-hematopoietic cells establishing local milieu that could promote induction or alleviate perpetuation of inflammation. Accordingly, serum levels of BAFF seem to be increased in several various autoimmune diseases, such as rheumatoid arthritis, Sjogren’s syndrome, SLE, and IBD [45,46,47,48]. Further studies demonstrated that the increased levels of serum BAFF were associated with increased levels of autoantibodies in these diseases and correlated with disease activity in most cases [45,46,47,48]. Notably, accumulating evidence suggests that BAFF may contribute to some B-cell malignancies such as non-Hodgkin’s lymphoma, chronic lymphocytic leukemia and multiple myeloma and that the role of BAFF in these malignancies may lie in its aberrant expression and production in these circumstances [49,50,51].

Given its role in systemic autoimmune disorders, drugs targeting BAFF, such as belimumab, atacicept, blisibimod and tabalumab, have recently become clinically approved and commercially available for treatment of inflammatory disorders. It is however important to address that albeit the monoclonal BAFF antibody belimumab was approved for SLE treatment, having a low incidence of organ damage and an excellent safety profile, most of the other BAFF inhibitors were much less effective in clinical trials. These results suggest redundancies in B-cell signaling pathways, and thus highlight the importance of better understanding of BAFF role in autoimmune diseases before future clinical trials are conducted [52,53].

## 3. BAFF in IBD

### 3.1. Role of B-Cells in IBD

Despite myriad of distinct biological pathways have been implicated in its pathophysiology, it is generally believed that IBD is a result of a maladaptive immune response to gut-resident commensal bacteria in a genetically susceptible host [54]. Inflammation in CD seems to be mainly driven by T_h_1 responses, whereas T_h_2 responses dominate the pathobiology of UC [55]. Nevertheless, additional lymphocytes, such as innate lymphoid cells and T_h_17 cells have also arisen as key players in the pathogenesis of IBD [56]. Specifically, abnormal pro-inflammatory CD4^+^ T-cell responses mediated by effector T_h_1, T_h_2, or T_h_17 cells disrupt homeostasis and causes IBD by outweighing anti-inflammatory CD4^+^ T-cell responses orchestrated by T regulatory (T_reg_) cells [57]. Even though T-cell system is predominant in studies concerning the IBD pathogenesis and therapeutic approach, emerging data suggest a role of B-cell lineage in IBD as well. Firstly, humoral homeostasis seems to be impaired in IBD. For instance, it has been shown that production of functional, dimeric immunoglobulin A (IgA) is impaired in patients with IBD [58]. As IgA exerts local anti-inflammatory effects by coating commensal bacteria after undergoing transepithelial translocation in gut, its depletion favors gut inflammation [59]. Moreover, B-cell expression of the pro-inflammatory cytokine IL-8, as well as production of mucosal IgG in gut are upregulated in IBD, thereby further promoting inflammation [60,61]. Accordingly, expression of both IL-8 and TLR-2 in IBD patients positively correlated with CD activity [61,62,63].

In murine IBD model, poorly regulated B-cells have been shown to exacerbate inflammation by blocking T_reg_ cell function [64,65]. Moreover, B-cells promote ileitis in UC by producing epithelial cell-specific autoantibodies [64,66]. Mucosal IgG in IBD can be directed against microbial elements, such as anti-saccharomyces-cerevisiae antibodies (ASCA) and anti-flagellin antibodies, or autoantigens, such as anti-neutrophil cytoplasmic antibodies (ANCA) and anti-epithelial antibodies [67,68]. Notably, the role of anti-granulocyte macrophage colony-stimulating factor (anti-GM-CSF) in IBD is fairly complex, yet the presence of anti-GM-CSF in the setting of IBD is associated with ileal phenotype and intricated behavior of the disease [69]. The latter observation is in line with preclinical data, as NOD2 KO mice treated with anti-GM-CSF antibodies develop transmural ileitis subsequent to NSAID exposure [69]. Alterations of the B-cell lineage in IBD are less obvious, and more complex for that matter, than those associated with derangement of T-cell system [70]. Acknowledgement of poor understanding of the complexity of B-cell responses in IBD allows us to evaluate recently failed therapeutic attempts in UC involving the CD20-targeting agent rituximab more critically [31]. Namely, negative outcomes of therapeutic approaches including rituximab should not discourage from considering B-cells as potential therapeutic target in IBD, especially since unresponsiveness to rituximab can be also observed in certain cases of B-cell-related autoimmune disorders such as rheumatoid arthritis (RA) and vasculitis [71,72]. For example, in certain group of patients, paradoxical pro-inflammatory manifestations can occur subsequent to rituximab administration [73,74]. While dysfunctional B-cell lineage can promote autoimmunity via autoreactive, long-lived plasma cells, regulatory B-cells can attenuate inflammation too. For instance, anti-CD20-treated mice deficient in peripheral B-cells failed to undergo spontaneous recovery and even developed chronic disease in a model of murine autoimmune encephalomyelitis [75,76]. In addition, B-cells may produce anti-inflammatory IL-10 but may also promote the anti-inflammatory effect of T_reg_ cells [77]. Even more perplexing is the fact that tissue resident plasma cells do not express CD20 and thus cannot be targeted by rituximab [78]. In the setting of IBD, in vitro experiment showed that plasma cells subset expanded in the mucosa of IBD patients and was resistant to rituximab-induced apoptosis [79]. Nevertheless, evidence of rituximab in IBD is conflicting, as some studies showed that rituximab could improve colonic inflammation, whereas case reports showed that rituximab could trigger colitis [80,81,82,83]. In fact, a retrospective cohort study showed that patients on rituximab have a sixfold increased risk of developing IBD compared to the general population [84]. Finally, a phase II randomized controlled trial, in which effects of rituximab on UC patients was assessed, showed no significant effect on inducing remission in moderately active UC not responding to oral steroids with possible short-term response that was not sustained [80].

### 3.2. Pathophysiological Background of BAFF in IBD

Even though BAFF is widely considered as a cytokine affecting B-cells primarily, and to a lesser extent T-cells, BAFF has been also shown to affect innate immunity by multiple effector arms [85]. Namely, it has been well documented that BAFF improves human monocyte survival, upregulates proinflammatory cytokine secretion, and positively regulates secretion of multiple costimulatory molecules [86]. These findings may be important for explaining the role of BAFF in IBD, as genome-wide association studies have highlighted the importance of host innate immune responses to microbes in the pathogenesis of IBD [83]. Single nucleotide polymorphisms associated with increased risk of developing IBD were identified in genes encoding microbial sensing and clearance, as well as integrating antimicrobial adaptive immune responses [87]. Accordingly, evidence suggests that macrophages and dendritic cells residing in gastrointestinal system have important interactions with the microbial environment, resolution of mucosal inflammation, proinflammatory tissue injury, and induction of adaptive immune responses [88,89,90]. Despite the presence of BAFF is most commonly associated with the induction of autoimmune inflammation, alternative function including induction of B regulatory cells that protect the intestinal mucosa from inflammatory injury has also been explored [91]. It was recently demonstrated that mice with DSS-colitis exhibit a persistent decrease in colonic CD5(+) regulatory B-cells (B_reg_), suggesting that persistent altered mucosal B-cell population caused by chronic gut inflammation may be involved in the pathogenesis of IBD [92]. These conclusions were corroborated in a small-sample clinical study, as UC patients had significantly reduced frequencies of CD5(+) B_reg_ in peripheral blood and intestinal tissues, accompanied by lower serum IL-10 levels [93]. In the same study, Mayo clinic scores, CRP, and ESR in UC patients negatively correlated with the frequency of B_reg_ and the IL-10 concentration. Nevertheless, as we further discuss, BAFF serum levels positively correlate with clinical disease activity and inflammatory biomarkers, thus indicating that the role of BAFF in IBD is primarily proinflammatory [94]. Finally, the strongest evidence indicating contribution of BAFF to IBD pathogenesis is data from colonic biopsies from UC and CD patients [94]. In colonic bioptates of both UC and CD patients, mRNA and BAFF protein expression were higher than in the control group. Furthermore, in inflamed regions of UC mucosa, upregulation of BAFF was predominant in mononuclear cells residing in lamina propria [94].

Finally, an important link connecting BAFF with IBD is the Nuclear Factor kappa-light-chain-enhancer of activated B-cells (NFκB), protein complex involved in control of transcription of DNA in almost all mammalian cells [95]. In response to various pro-inflammatory stimuli, NFκB activates and leads to in the increased expression of adhesion molecules and chemokines by endothelial cells and in the tissue, thus favoring the recruitment and activation of effector immune cells [96]. Multiple line of evidence suggests important role of NFκB activation in IBD pathogenesis [97,98]. Activation of NFκB is detected in both epithelial cells and macrophages from IBD patients and relates to the intensity of inflammation [99]. Furthermore, administration of antisense phosphorothioate oligonucleotides to the p65 subunit of NFκB has been shown to suppress colitis in mice model of TNBS-induced colitis [100]. Finally, effectiveness of corticosteroid treatment in IBD flares is in part owing to steroid-induced decrease of NFκB activation [101]. As BAFF is capable of activating NFκB in lymphoid and myeloid cells via both canonical and non-canonical pathway, thus promoting intestinal inflammation, it is plausible that NFκB is the missing link between BAFF and IBD [102].

The presented results, alongside the fact that fecal BAFF was increased in both adults and children with inactive disease, it could be speculated that BAFF is not only related to the disease flares but also to the pathogenetic substrate of IBD. However, whether the alterative B-cells and antibodies production in IBD are related to high levels of BAFF remain unclear. In order to answer this question, longitudinal studies are warranted.

### 3.3. Clinical Implications of BAFF in IBD

In light of the prominent role of BAFF in B-cells and autoimmunity, Zhang et al. sought to explore the putative role of BAFF in management of IBD [94]. The principal aim of that pivotal study was to determine the value of BAFF to discriminate patients with IBD from healthy controls and patients with IBS by measuring BAFF serum and fecal levels, as well as its mucosal expression. An additional aim was to establish whether there is a correlation between BAFF and disease activity in IBD patients. It was demonstrated that BAFF expression is increased in serum, feces and colonic mucosa of patients with IBD when compared to controls. Moreover, in comparison to IBS patients, significantly higher fecal BAFF concentrations were observed in patients with IBD, regardless of disease activity, with fecal BAFF concentrations in IBS patients being identical to those of healthy controls. In fact, for BAFF fecal levels above cut-off of 325 pg/mL, respectively. Even higher sensitivity (90%) was observed for discrimination between active IBD and IBS/healthy controls, which is in line with strong positive correlations observed between BAFF and disease activity, TNF-α and IL-1β in patients with UC. The discriminative power of serum BAFF had comparable specificity (93%), but markedly lower sensitivity (55%) than fecal BAFF. Nevertheless, it appears that, following the reduction in disease activity, BAFF serum levels return to values similar to that of healthy subjects rapidly, unlike BAFF levels in feces which persist much longer. Thus, it is plausible that serum BAFF may be utilized in monitoring the disease activity.

Clinical distinction between IBD and IBS is of utmost importance, as these two conditions can present similar symptoms, but have very different underlying pathophysiology and, more importantly, severity of consequences [103]. In the absence of additional symptoms indicating on IBD, such as rectal bleeding and systemic illness, it is very challenging to distinguish between the two. On the other hand, although it is a common practice, it is not cost-effective to use colonoscopy as a part of diagnostic algorithm in the workup of patients suspected of having IBS [104,105]. Moreover, colonoscopy is associated with serious complications such as bleeding and perforations, adverse events related to the anesthesia, and increased discomfort of patients [106]. Hence, it would be beneficial to use sensitive and specific biomarker for IBD/IBS differentiation. So far, calprotectin has been used for this purpose with relative success owing to sufficient sensitivity, yet what calprotectin lacks is adequate specificity [107].

In this sense, Fu et al. compared the efficacy of fecal BAFF, calprotectin and fecal occult blood test (FOBT) to find the “best non-invasive marker” [108]. The study showed that for discriminating IBD from IBS, fecal BAFF ≥ 227.3  pg/mL yielded 84% sensitivity and 100% specificity, calprotectin ≥ 50  µg/g yielded 76% sensitivity and 93% specificity whereas FOBT yielded 65% sensitivity and 93% specificity. Moreover, combination of BAFF with calprotectin yielded 94% sensitivity and 93% specificity, thus increasing the accuracy of differential diagnosis. Notably, fecal BAFF concentration exhibited stronger correlation with endoscopic inflammatory score in comparison to calprotectin in both UC (r = 0.69, *p*  <  0.0001 vs. *r*  =  0.58, *p*  <  0.0001) and CD (*r*  =  0.58, *p*  <  0.0001 vs. *r*  =  0.52, *p*  =  0.0003). Accordingly, a separate study confirmed that fecal BAFF is more sensitive and specific in predicting UC activity and severity than fecal calprotectin [109]. On a separate note, neither fecal BAFF nor calprotectin showed significant correlation with Crohn’s Disease Activity Index (CDAI) in CD patients, yet they both showed correlation with Mayo score in patients with UC (*r* = 0.415 and 0.365, respectively). Furthermore, Xie et al. explored whether BAFF can discriminate patients with IBD and malignancy from other gastrointestinal diseases among population of patients presenting with abdominal discomfort [110]. It was demonstrated that fecal BAFF was able to accurately distinguish patients with either IBD or tumor from patients without any of these, giving a sensitivity of 85% and specificity of 91%. On the other hand, BAFF was also able to discriminate IBD from patients without it with sensitivity of 89% and specificity of 77%. An important finding of this study is that BAFF was found to be temperature-stable for 7 days and equally distributed in the feces, thus implying that for BAFF measurement no specific storage conditions are required and only a small number of samples are needed to accurately measure it. Altogether, the above-noted results imply that BAFF could be used as a complementary biomarker in diagnostic workup of patients with suspected IBD and that it may also be utilized as a sensitive surrogate for assessment of endoscopic inflammation in IBD. Nevertheless, further research is warranted to elucidate in deep the values of fecal BAFF in IBD clinical scenery, such as whether fecal BAFF can be used to predict relapse in IBD patients in remission, as shown in calprotectin [111].

Usefulness of BAFF in the setting of IBD has been explored in pediatric population as well. The problem of IBD diagnosis and follow-up represents an even bigger challenge children then among the adults. Firstly, colonoscopy is not easily accepted by children nor caregivers compared to adults [112]. Moreover, currently no consensus was established with regard to the definition of remission in pediatric IBD. The term “clinical remission” is the most widely accepted term defined by composite scores based on clinical parameters (abdominal pain, rectal bleeding, stool frequency and consistency, number of stools per 24 h, nocturnal stools, activity level, weight). Notably, laboratory findings (ESR and albumin) are used in the wPCDAI score exclusively. As multiple studies showed that there is a discrepancy between the clinical remission and the laboratory, endoscopic and histologic findings, there is a need for a comprehensive approach, including clinical, imaging, histologic and laboratory parameters [113,114,115]. In a recent prospective study, Fodor et al. found no differences in serum BAFF between IBD, IBS, and healthy group, yet they found that fecal BAFF was higher in the IBD group in comparison to IBS and healthy group [116]. In comparison of different types of IBD, it was shown that BAFF is higher in pediatric patients with UC compared to CD patients. ROC curve analysis for fecal BAFF showed that with cut-off of 16 pg/mL, sensitivity and specificity for discrimination between IBD and IBS in pediatric population is 51% and 93%, respectively. The observed lack of sensitivity could be owing to the fact that only mild cases of IBD were included and limited number of participants. Finally, as fecal BAFF was the highest in patients with increased calprotectin levels, the authors proposed that fecal BAFF may be a promising marker in the evaluation of the remission status in pediatric IBD.

Of note, in a recent study, Andreou et al. explored whether serum BAFF can serve as a potential prognostic indicator of therapeutic response to Infliximab treatment in CD [117]. Baseline serum BAFF values were significantly higher in the responders’ group in comparison to non-responders to infliximab (610.03 ± 167.55 pg/mL vs. 267.09 ± 107 pg/mL), with both groups having higher serum levels than healthy controls (128.16 ± 70.10 pg/mL). Interestingly, after treatment with infliximab, serum BAFF levels reduced among responders, and increased among non-responders, thus reaching similar values (333.40 ± 178.75 pg/mL vs. 438.58 ± 180.01 pg/mL, *p* = 0.130). Unfortunately, the authors did not to interpret these findings from pathophysiological standpoint. The reduction of BAFF in responders is in accordance with the available data that suggests BAFF levels correlate with disease activity, yet the observed increase among non-responders is rather perplexing and warrants further research.

## 4. Future Directions and Conclusions

In conclusion, available data suggests that BAFF may become a biomarker of IBD in the upcoming years. An ideal biomarker for IBD should be easy to perform, noninvasive or minimally invasive, cheap, rapid, and reproducible. In addition, the putative diagnostic biomarker should be able to identify individuals at risk of developing the disease and reflect the disease activity. Notably, importance of biomarkers in IBD approach is crucial, since colonoscopy as a diagnostic method is burdened by adverse effects, discomfort, and insufficient cost-effectiveness and should therefore be reserved for patients with high probability of disease exclusively. Since BAFF expressed favorable characteristics with regard to sensitivity and specificity in discriminating between IBD and IBS, it is plausible that it could be used as a part of diagnostic algorithm of IBD in the upcoming future (Table 1). As fecal calprotectin has high sensitivity but markedly low specificity, and fecal BAFF exerts variable sensitivity but consistently high specificity, it would be useful to combine these two biomarkers in diagnostic approach to IBD. Although Fu et al. demonstrated incremental diagnostic value when these two are combined, since all currently available studies were conducted on a limited sample, further large-scale studies are warranted in order to confirm these presumptions. Apart from diagnostic role, as serum BAFF was shown to correlate with disease activity, returning to values similar to healthy subjects in inactive IBD, its values may be used as supplemental indices of disease activity, especially in pediatric population which is burdened by lack of precise definition of remission. In this notion, fecal BAFF was also found to correlate with indices of inflammation in IBD, but with BAFF levels returning to values of healthy subjects more slowly in comparison to serum levels. Finally, respecting the pathogenetic role of BAFF in IBD and driven by the clinical success of BAFF inhibitors in multiple autoimmune diseases, it is reasonable that targeting BAFF could be a novel and unexplored strategy in IBD treatment.

## Figures and Tables

**Table 1:** Clinical studies concerning the role of BAFF in IBD.

Study	Population	Purpose	Results
Zhang et al. [94]	78 UC 37 CD 12 IBS 44 HC	correlation between serum/fecal BAFF and UC) disease severity (Mayo score) discriminative value of fecal BAFF (IBD from IBS/healthy controls) expression and distribution of BAFF in intestinal mucosa	positive correlation between fecal BAFF and Mayo score (*r* = 0.425; *p* = 0.017) for fecal BAFF > 325 pg/mL: 84% sensitivity and 96% specificity higher expression of BAFF in both UC (*p* = 0.003) and CD (*p* = 0.045)
Fu et al. [108]	49 UC 44 CD 27 IBS 26 HC	discriminative value of fecal BAFF, FOBT, calprotectin (IBD from IBS) fecal BAFF and calprotectin correlation with endoscopic inflammatory score	fecal BAFF ≥227.3 pg/mL: 84% sensitivity and 100% specificity calprotectin ≥50 µg/g: 76% sensitivity and 93% specificity FOBT: 65% sensitivity and 93% specificity BAFF + calprotectin: 94% sensitivity and 93% specificity BAFF vs. calprotectin in UC: r = 0.69, *p* < 0.0001 vs. *r* = 0.58, *p* < 0.0001 (correlation with endoscopic inflammatory score)
Hussein et al. [109]	50 UC *	predictive value of fecal BAFF vs. calprotectin for disease activity and severity in UC	Disease activity ○ Fecal BAFF > 47 μg/g: 97.5% sensitivity and 100% specificity ○ Calprotectin > 50 μg/g: 90% sensitivity and 90% specificity Disease severity ○ Fecal BAFF (r = 0.897, *p* < 0.001) ○ Calprotectin (r = 0.750, *p* < 0.001)
Xie et al. [110]	148 ** 44 HC	discriminative value of fecal BAFF (IBD/malignancy from healthy control)	Fecal BAFF > 219.5 pg/g: 85% sensitivity and 91% specificity BAFF and calprotectin: 92% sensitivity and 61% specificity
Fodor et al. [116]	32 CD 16 UC 10 IBS 26 HC	discriminative value of fecal BAFF (IBD from IBS in pediatric population)	fecal BAFF > 16 pg/mL: 51% sensitivity and 93% specificity
Andreou et al. [117]	112 CD 164 HC	BAFF as a potential prognostic indicator of therapeutic response to Infliximab treatment in CD	Serum BAFF in responders vs. non responders to infliximab: ○ Baseline: 610.03 ± 167.55 pg/mL vs. 267.09 ± 107 pg/mL ○ After treatment: 333.40 ± 178.75 pg/mL vs. 438.58 ± 180.01 pg/mL, *p* = 0.130

* 40 with active UC and 10 with inactive UC ** 13 with gastric polyps, 47 gastritis, 11 peptic ulcer, 21 gastric cancer, 11 colorectal polyps, 12 UC, 16 CD, 17 colorectal cancer. Abbreviations: CD: Crohn’s disease; UC: ulcerative colitis; HC: healthy controls; IBS: irritable bowel syndrome; BAFF: B-cell activating factor; IBD: inflammatory bowel disease.

## Data Availability

Not applicable.
